# Supplementary material for: A puzzling insensitivity of magnon spin diffusion to the presence of 180-degree domain walls
Source: Nat Commun. 2023 Apr 26;14:2393. doi: 10.1038/s41467-023-38095-3 (PMC10133301; doi:10.1038/s41467-023-38095-3)
Supplement: Supplementary file 1 — Supplementary information [file 41467_2023_38095_MOESM1_ESM.pdf]

Supporting Information

SUPPLEMENTARY NOTE 1: DETERMINATION OF THE MAGNETIZATION MAP FROM THE SCANNING NV CENTER IMAGE

The reconstruction of the magnetization is performed using a machine learning approach (manuscript in preparation). This approach takes the magnetic field image from the NV measurement and generates a mapping to the source, in this case the magnetization of the material. The machine learning loss function is then generated by forward propagating the reconstructed magnetization back into a magnetic field image, which is compared to the original magnetic image. This approach ensures that the reconstructed magnetization image is a valid solution to the magnetic field image that was measured.

For the reconstruction shown in Figure 1B in the main text, we assumed that the local magnetization was oriented along either the $[1\bar{1}0]$ or $[\bar{1}10]$ direction, consistent with the magnetization hysteresis measurements in Figure 1A. The correlation between the magnetization map and the stray-magnetic-field map is perhaps not obvious by eye, because a strong magnetic stray field is produced only at domain edges that are perpendicular to the direction of magnetization, not the parallel edges. It is for this reason that the orientation of the stripe-like features in the stray-field map is different than in the magnetization map. As a test, we have also explored the results of the reconstruction algorithm assuming that the local magnetization is oriented along the [010] or $[0\bar{1}0]$ directions, along [110] or $[\bar{1}\bar{1}0]$, or along [100] or $[\bar{1}00]$ (see Supplementary Figure 1), but these assumptions were not able to reproduce the measured stray-field maps with the same low error as the result in Figure 1B in the main text.

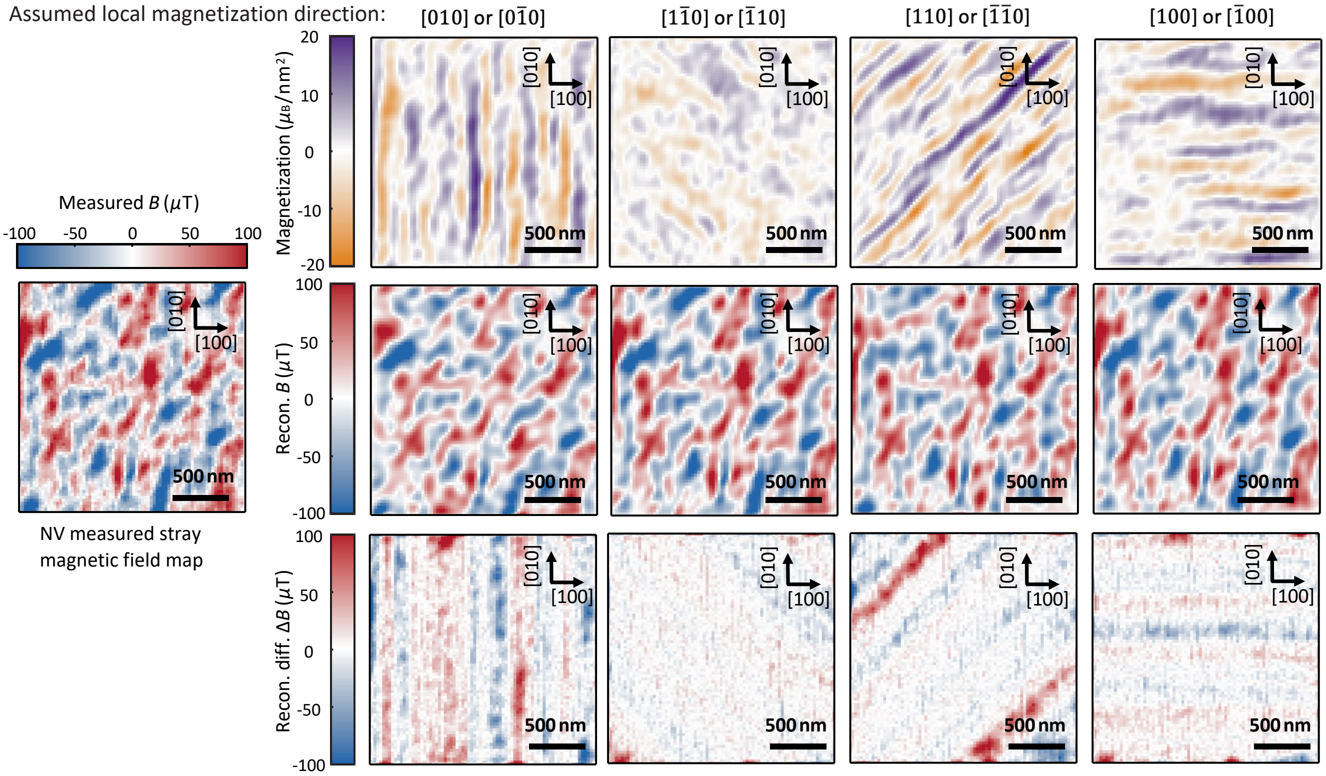

Supplementary Figure 1. Comparison of the magnetization reconstruction assuming different local magnetization directions. The far left panel is the magnetic field measured by the NV center. The top row is the magnetization reconstruction by the machine learning algorithm with an assumed local magnetization direction along [010] or $[0\bar{1}0]$, along $[1\bar{1}0]$ or $[\bar{1}10]$, along [110] or $[\bar{1}\bar{1}0]$, or along [100] or $[\bar{1}00]$ (from left to right). The middle row is the reconstructed magnetic field from the magnetization above it. The bottom row is the difference between the reconstructed magnetic field and the measured magnetic field.

SUPPLEMENTARY NOTE 2: MICROMAGNETIC SIMULATIONS

The micromagnetic simulations are performed using the open-source software MuMax3 [1]. A 2D system with 1000 $\times$ 1000 discretized cells is considered, and the size of each cell is 2 nm $\times$ 2 nm, which is significantly smaller than the magnetic exchange length of the MAFO film, $l_{ex} = \sqrt{A_{ex}/0.5\mu_0 M_s^2} \approx 20$ nm. In this formula, the saturation magnetization $M_s = 9.549 \times 10^4$ A/m is from our own measurements; the exchange constant $A_{ex} = 2.22 \times 10^{-12}$ J/m is estimated based on the Curie temperature and the lattice parameters of MAFO [2, 3] and is consistent with typical values for spinel ferrites (for example, A_{ex} for cobalt ferrites is 4×10^{-12} J/m [4]). According to a previous study [5], our coherently-strained (001) MAFO thin films grown on (001) $MgAl_2O_4$ substrates have a large easy-plane anisotropy within the x-y plane, $E_{ep} = K_e m_z^2$ (arising possibly due to the epitaxial strain), as well as a modest biaxial in-plane cubic anisotropy with the crystallographic [110] and [1$\bar{1}$0] directions as the easy axes, $E_{bi} = K_{bi} m_x^2 m_y^2$. Here the anisotropy coefficients $K_e = 6.684 \times 10^4$ J/m^3 and $K_c = 477.5$ J/m^3; and m_i ($i = x, y, z$) are the components along the three Cartesian axes of the unit vector corresponding to the magnetization (e.g., $+x \parallel$ [110]). In the simulation, these two anisotropies are set to exist in all cells of the system. According to the magnetic hysteresis loop in Figure 1a of the main text, the [1$\bar{1}$0] axis is slightly easier than the [110] axis. To simulate this, we introduce 500 circular pinning sites with a radius of 10 nm that are randomly distributed in the system (see Supplementary Figure 2a). In each pinning site, there exists an additional uniaxial anisotropy along the [1$\bar{1}$0] axis, $E_{uni} = -K_{uni}(\mathbf{m} \cdot \mathbf{u})^2$, where $\mathbf{u}$ is a unit vector along the [1$\bar{1}$0] axis, with $K_{uni} = 4000$ J/m^3. The magnetization in these pinning sites is randomly initiated (see Supplementary Figure 2b). The equilibrium magnetic domain pattern is obtained by solving the Landau-Lifshitz-Gilbert (LLG) equation using the fourth-order Runge-Kutta method with a time interval of 10 fs. Periodic boundary conditions are applied along both the x and y axes. The effective magnetic field, which drives the evolution of the initial magnetization distribution to equilibrium, is the sum of magnetic stray field, the Heisenberg exchange coupling field, and the fields arising from the magnetic anisotropies mentioned above. The effective Gilbert damping coefficient α is set to 0.004, as extracted from FMR measurement performed on the 6 nm film.

When we simulate the zero-field magnetic configuration after the application of a saturating magnetic field along the [110] axis, the results indicate the formation of stripe domains oriented along [1$\bar{1}$0] with spins aligned along the same axis and with 180° domains walls separating the domains (see Supplementary Figure 3). In this model, the pinning sites work as nucleation sites for the formation of the stripe domains. The results are qualitatively consistent with the NV-center image in Figure 1b of the main text. The simulated stripe pattern is more ordered than the experimental result, but this may be related to differences in the strength of the pinning sites and the assumption of periodic boundary conditions in the simulation.

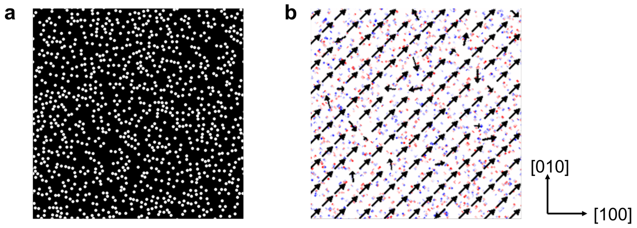

Supplementary Figure 2. (a) The location of the 500 randomly distributed pinning sites, which are shown as the white dots and have a radius of 10 nm. (b) The assumed initial magnetization distribution after application of a magnetic field in the [110] direction and then removal of the magnetic field. The magnetization vectors are randomly oriented inside the pinning sites, and are along the [110] direction elsewhere.

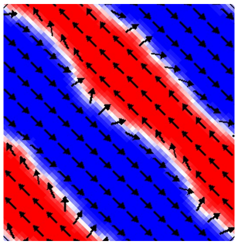

Supplementary Figure 3. Domain pattern in the demagnetized state obtained via micromagnetic simulations.

SUPPLEMENTARY NOTE 3: ADDITIONAL FIELD DEPENDENCE DATA

Field Dependence with Different Sweeping Angles

We have measured first-harmonic nonlocal resistances with the external field H_{ext} swept along different in-plane directions ϕ with respect to the Pt wires, as shown in Supplementary Figure 4. This figure shows a wider range of field angles and field strengths than Figure 3 in the main text. At high fields, we observe a slight decrease in the first-harmonic signals due to an increasing Zeeman gap. In the low field regime, $R_{1\omega}$ converges to the same value for a given sample regardless of the angle of the sweeping field. These results confirm the robustness of our observations near zero field.

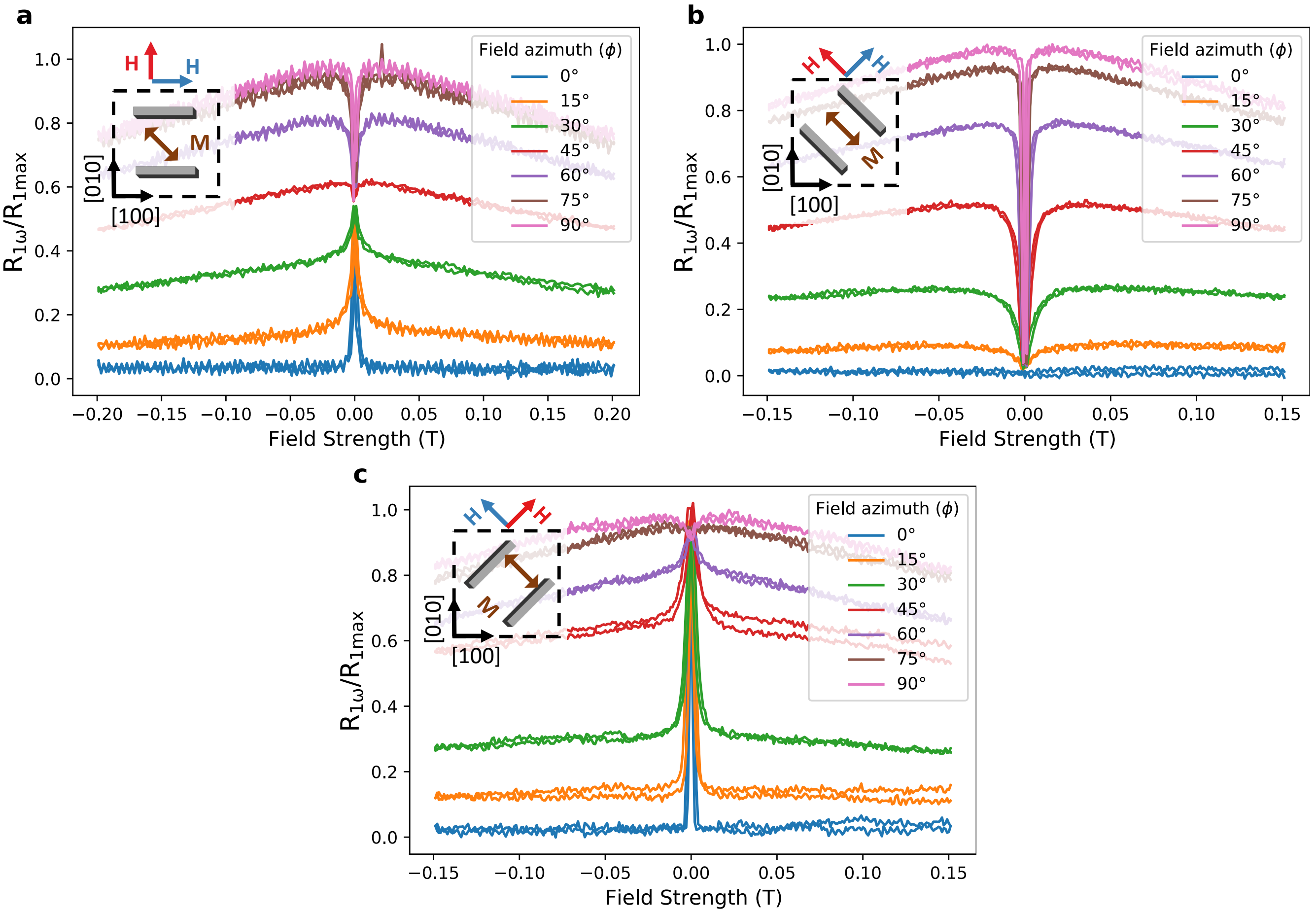

Supplementary Figure 4. Normalized first-harmonic nonlocal resistances for for the same 6 nm MAFO samples discussed in the main text with wire separations of 1 μm, with Pt wires along (a) [100], (b) [1$\bar{1}$0], and (c) [110] axes. The lines with different colors correspond to scans with the field H_{ext} swept along different angles ($0^\circ \leq \phi \leq 90^\circ$) with respect to the Pt wires.

Field Dependence with Different Spacings

Here we present the field dependence of first-harmonic nonlocal resistance with different spacings between the injector and detector Pt bars. The signal-to-noise ratio decreases as the spacing increases, but the trend remains the same.

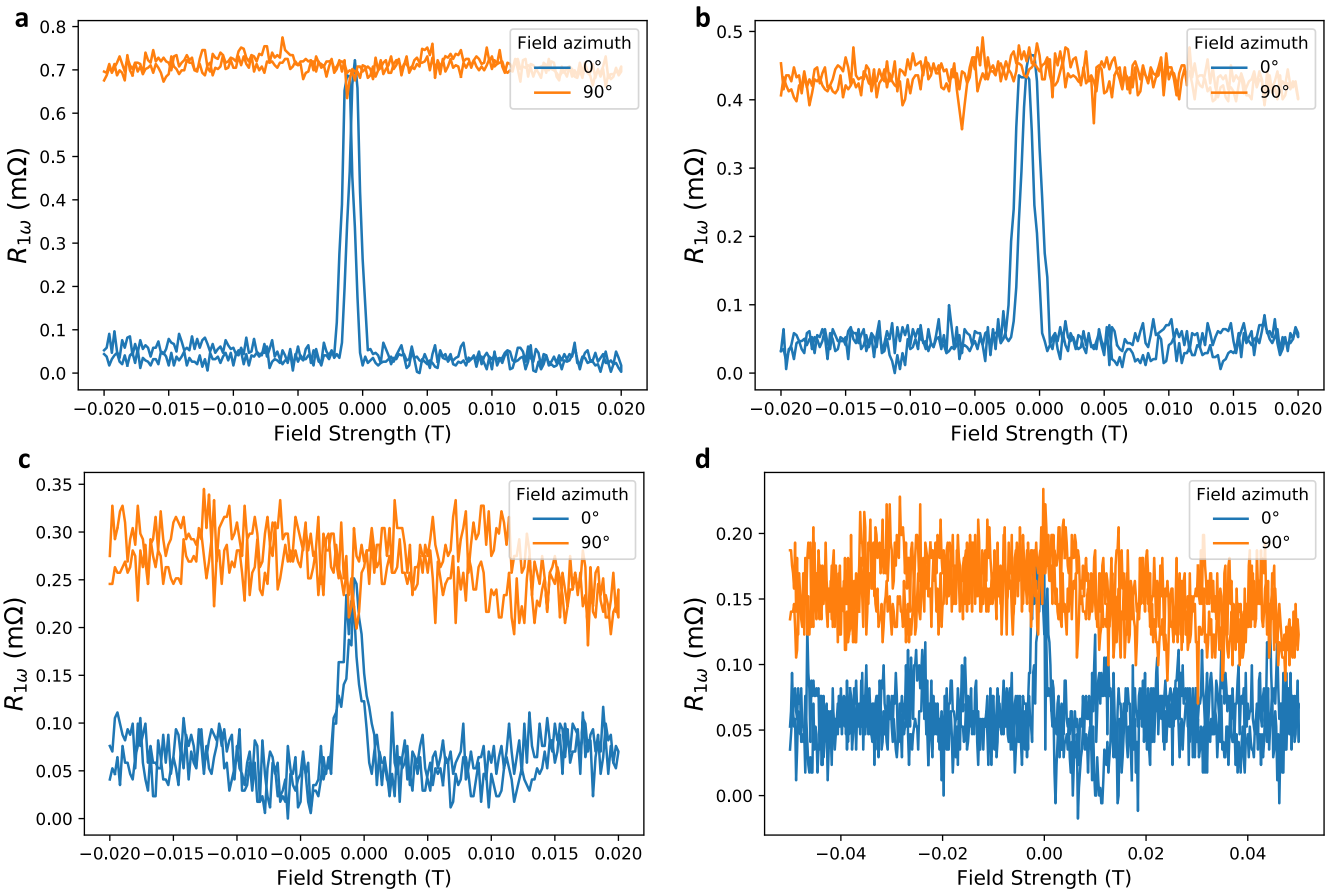

Supplementary Figure 5. First-harmonic nonlocal resistances for same 6 nm MAFO samples discussed in the main text with a wire separation of (a) 1.8 μm, (b) 2.2 μm, (c) 3.4 μm and (d) 4.2 μm.

Field Dependence for a 10 nm MAFO Film

For 10 nm MAFO samples grown under similar conditions as the 6 nm film highlighted in the main text, we observed similar magnetic hysteresis loops, as shown in Supplementary Figure 6 below. With this 10 nm MAFO film, we measured similar dependence for $R_{1\omega}$ as a function of magnetic field, shown in Supplementary Figure7 below.

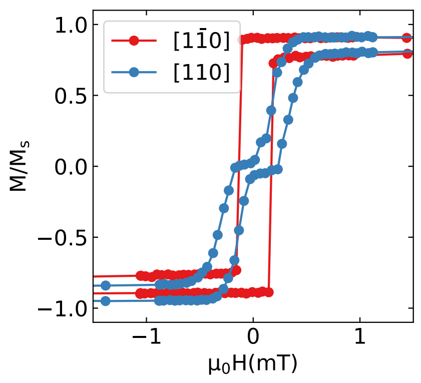

Supplementary Figure 6. Magnetic hysteresis loops measured with a SQUID magnetometer for a 10 nm MAFO film, which was grown under the same conditions as the 6 nm film used for nonlocal spin transport measurements. The magnetic field is swept along the in-plane [1$\bar{1}$0] and [110] axes.

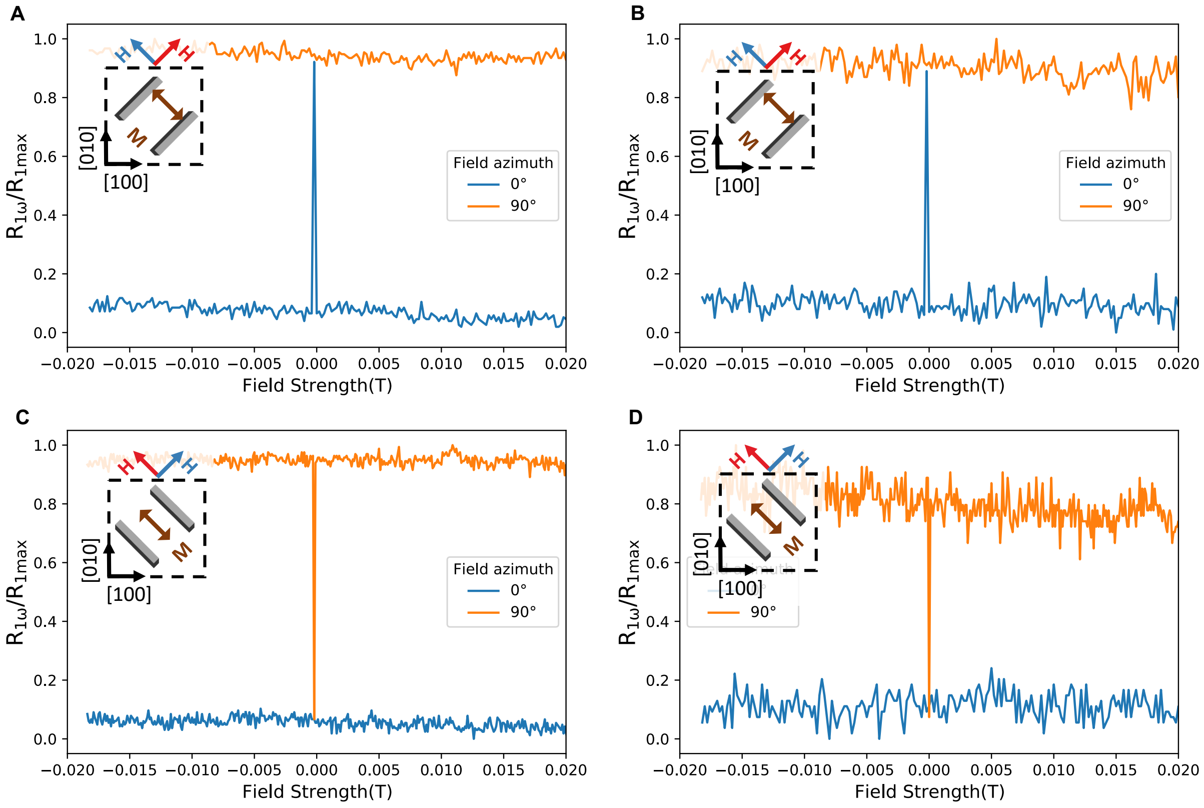

Supplementary Figure 7. Normalized first harmonic nonlocal resistance, measured on a 10 nm MAFO film with wire separations of (a,c) 0.8 μm and (b,d) 1.2 μm with the Pt wires along (a,b) [110], and (c,d) [1$\bar{1}$0] axes. The orange lines correspond to scans for magnetic field perpendicular to the Pt wires and the blue lines to field parallel to the Pt wires.

SUPPLEMENTARY NOTE 4: INVESTIGATING THE POSSIBILITY OF MAGNON-DRIVEN LONG-RANGE MOTION OF DOMAIN WALLS

To test whether the efficient transmission of angular momentum we observe near zero magnetic field might be associated with long-range translation of domain walls driven by magnons, we performed NV center imaging before and after applying in-situ current pulses. Supplementary Figure 8 shows the results for 0.2 mA and −0.2 mA pulses for a set of Pt wires with 6 μm spacing. The domain pattern remains unchanged at these current levels, without any indication of domain-wall translation. From this we conclude that the domains are well-pinned on the time scale of the measurement.

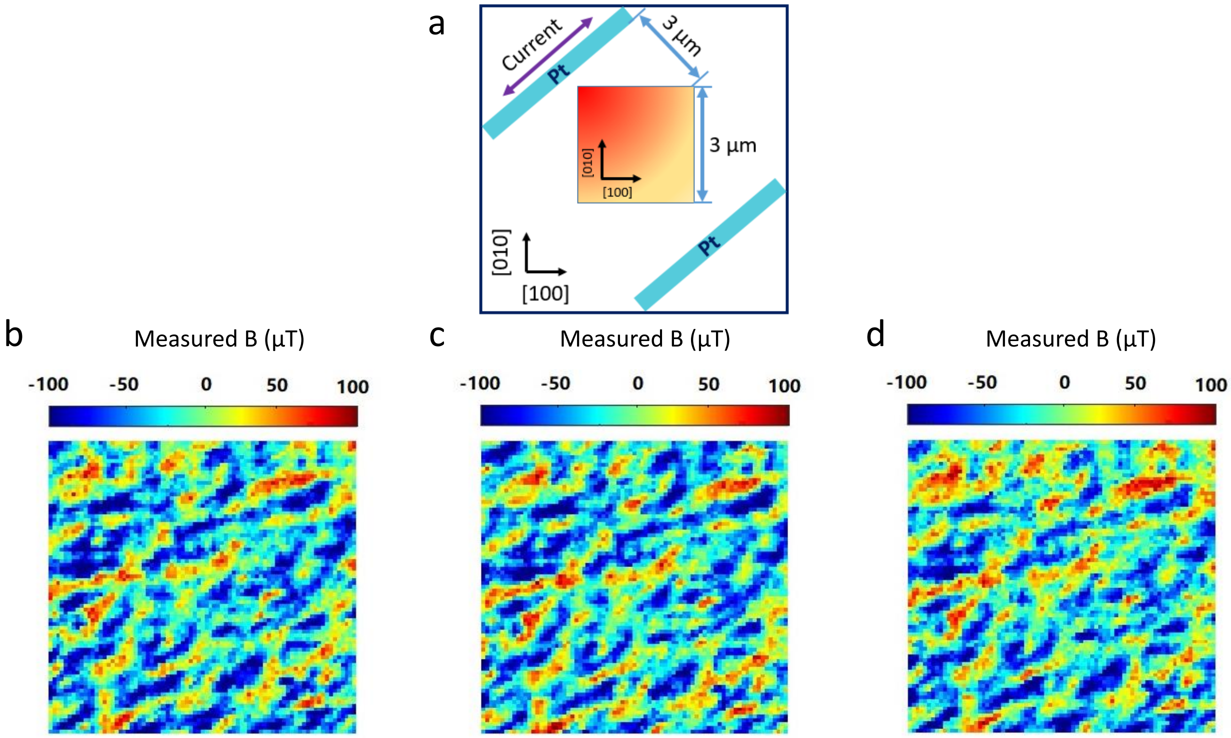

Supplementary Figure 8. (a) Schematic diagram of the measurement geometry. (b-d) NV-center microscopy images due to the pattern of magnetic domains in the 6 nm MAFO film (b) before and after applying 0.3 s long current pulses with amplitude 0.2 mA and −0.2 mA.

As another test of whether the nonlocal first-harmonic voltage $V_{1\omega}$ near zero magnetic field could be associated with angular momentum carried by long-range translation of domain walls, we tested whether the signals have any nonlinear dependence on current amplitude. Given that the domain walls are pinned, we anticipated that any overall translation should be suppressed for small current amplitudes and but then increasingly activated with increasing current amplitude. Supplementary Figure 9 shows the first-harmonic signal amplitude near zero applied magnetic field as a function of current amplitude for a pair of Pt wires separated by 2.2 μm, which is well above the average size of domains near zero field (hundreds of nanometers). We do not observe any deviation from a simple linear dependence.

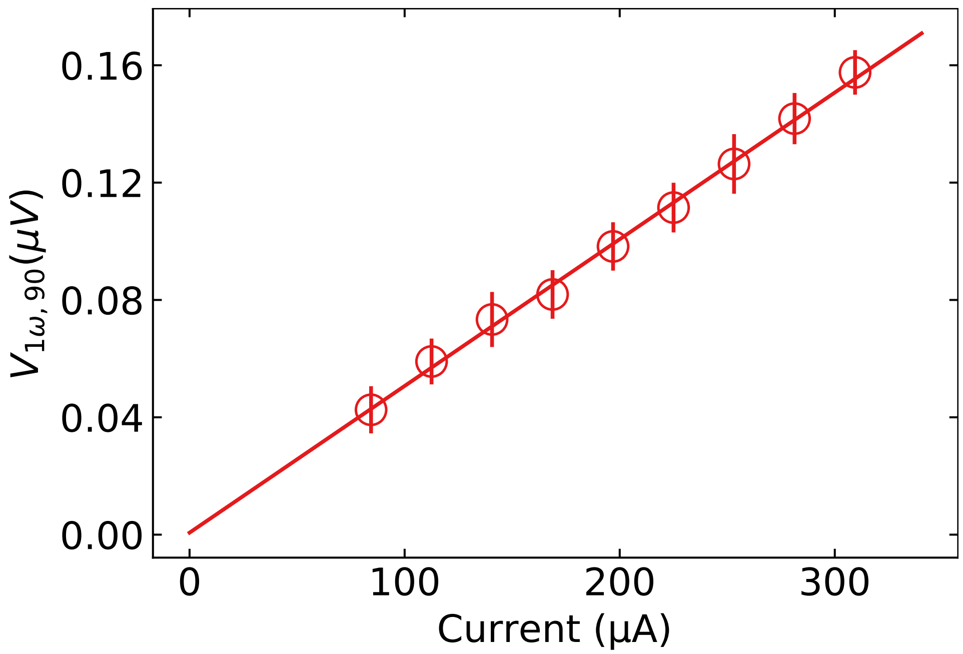

Supplementary Figure 9. Current dependence of averaged low-field (<10 mT) first-harmonic voltage for a 6 nm MAFO film with Pt wires along the [110] axes, separated by a distance of 2.2 μm. The external magnetic field is swept perpendicular to the Pt wires.

SUPPLEMENTARY NOTE 5: SCANNING NV CENTER MAGNETOMETRY SETUP

Supplementary Figure 10 shows the experimental setup of the scanning NV center magnetometry, with the reference angles indicated. The x direction in the figure corresponds to the $[1\bar{1}0]$ crystal axis.

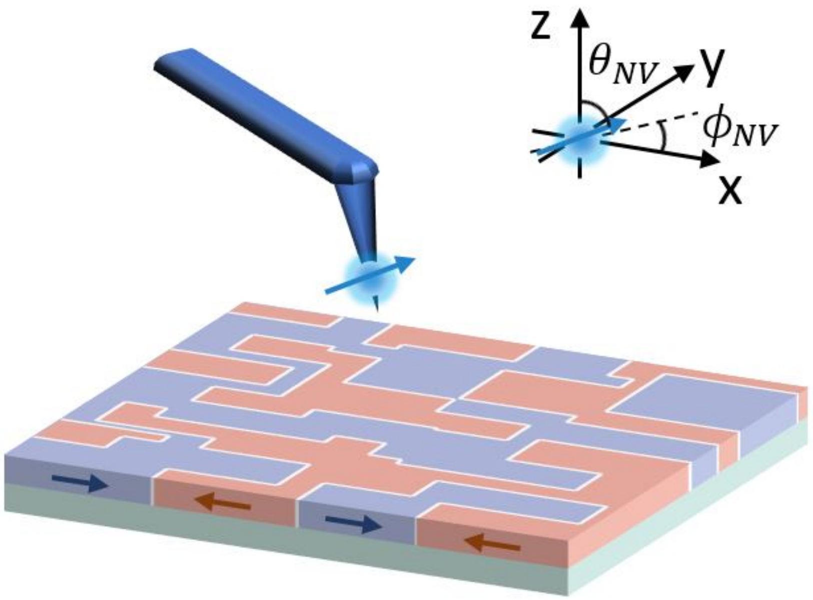

Supplementary Figure 10. Schematic drawing of scanning NV center magnetometry

SUPPLEMENTARY NOTE 6: DAMPING COEFFICIENT FROM FMR MEASUREMENTS

The MAFO sample was positioned in a downward-facing orientation on a coplanar waveguide, allowing the Oersted field produced by the waveguide to stimulate GHz frequency ferromagnetic resonance (FMR). FMR spectra were measured on the 6 nm MAFO sample at fixed frequencies ranging from 7 GHz to 18 GHz by sweeping the applied magnetic field (as shown in Supplementary Figure 11(a)). We extracted the linewidths of the FMR spectra and plot them as a function of frequency in Supplementary Figure 11(b). The dependence of the linewidth (ΔH_{hwhm}) on frequency (f) follows the Equation:

$$\Delta H_{\mathrm{hwhm}} = \Delta H_0 + \frac{h}{g\mu_0\mu_{\mathrm{B}}}\alpha f \tag{1}$$

where ΔH_0 is the zero-frequency linewidth, h is the Planck constant, g is the Lande g-factor (2.05), μ_0 is the permeability of free space, μ_{B} is the Bohr magneton, and α is the Gilbert damping coefficient. By applying this linear fit to the data, we obtained a damping coefficient of 0.004 for the film.

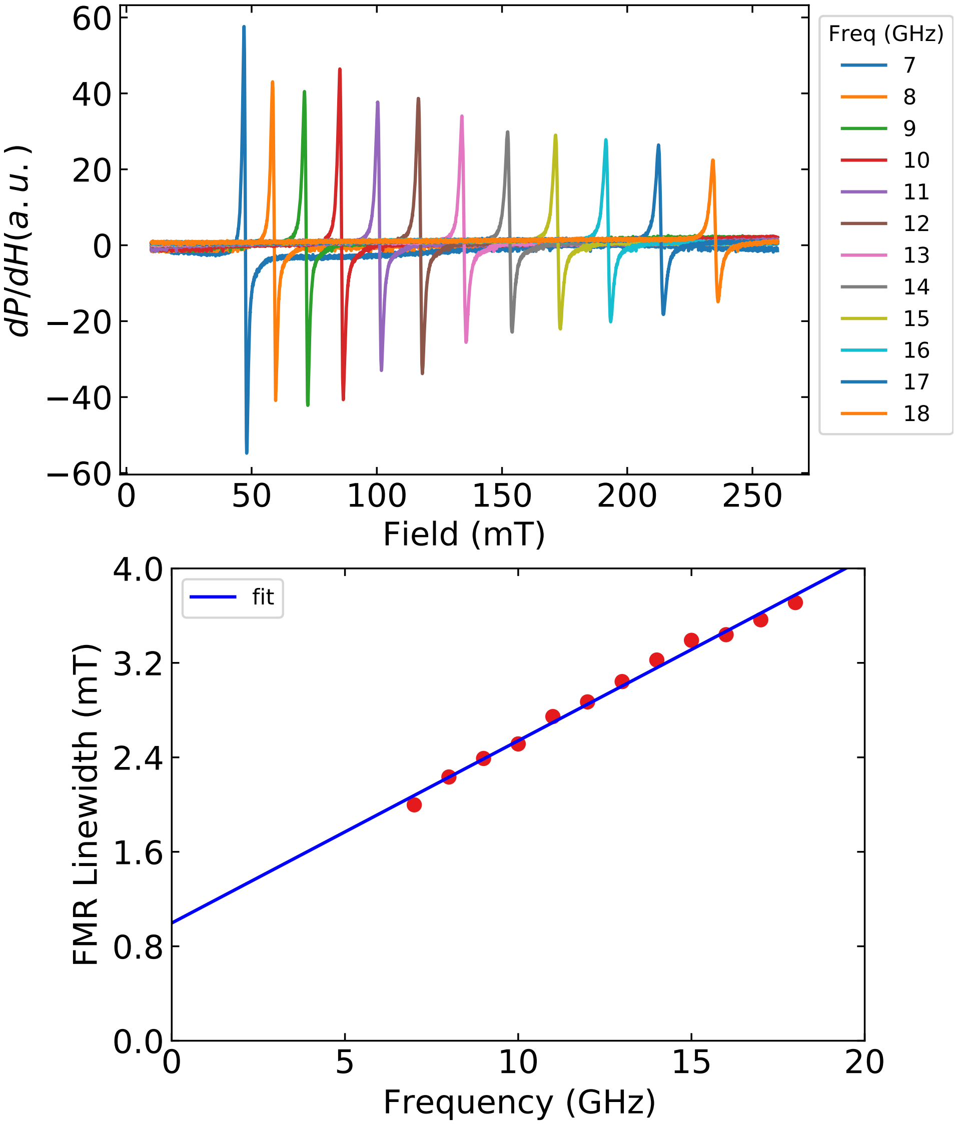

Supplementary Figure 11. (a) FMR spectra for 6 nm MAFO film at frequencies ranging from 7 GHz to 18 GHz (b) Frequency dependence of FMR linewidth

SUPPLEMENTARY NOTE 7: THEORETICAL MODEL

Ferrimagnetic model

The system can be modeled by a bipartite ferrimagnet which has two sublattices with unit spin vectors $\boldsymbol{n}_A$, $\boldsymbol{n}_B$. A convenient way to describe such a system is reparametrizing the two sublattice field by a staggered field $\boldsymbol{n} = (\boldsymbol{n}_A - \boldsymbol{n}_B)/2$ and $\boldsymbol{m} = \boldsymbol{n}_A + \boldsymbol{n}_B$. In terms of these new fields, the Lagrangian density is given by [6]

$$\mathcal{L} = -\mathcal{S}\dot{\boldsymbol{n}} \cdot (\boldsymbol{n} \times \boldsymbol{m}) - s\boldsymbol{a}(\boldsymbol{n}) \cdot \dot{\boldsymbol{n}} - \mathcal{E}(\boldsymbol{m}, \boldsymbol{n}). \tag{2}$$

Here, the first two terms describe the kinetic energy of spins, where $\mathcal{S} = (s_A + s_B)/2$ and $s = (s_A - s_B)/2$ with $s_{A/B}$ being the spin angular momentum density on the A/B sublattice, $\boldsymbol{a}(\boldsymbol{n})$ is the vector potential for the magnetic monopole. The total energy $\mathcal{E}(\boldsymbol{m}, \boldsymbol{n}) = \frac{a}{2}\boldsymbol{m}^2 + \mathcal{U}(\boldsymbol{n})$ with

$$\mathcal{U}(\boldsymbol{n}) = \frac{A}{2}(\partial_i \boldsymbol{n})^2 + \frac{K_c}{2} n_x^2 n_y^2 + \frac{K_e}{2}(n_z)^2 - \frac{K_{uni}}{2}(\boldsymbol{n} \cdot \boldsymbol{u})^2. \tag{3}$$

Here, a is the homogeneous exchange, the first term in $\mathcal{U}(\boldsymbol{n})$ stands for inhomogeneous exchange energy, the second and third term respectively describe the biaxial easy-axis and hard-axis anisotropy, and the last term represents an effective uniaxial anisotropy induced by homogeneously distributed nucleation sites, with the easy axis along the direction $\boldsymbol{u} = [1, \bar{1}, 0]$. Here, the hierarchy of the scales respects $K_e \gg K_{uni} \gg K_c$. According to numerical simulations, additional nucleation sites with a size similar to the domain-wall width are necessary for inducing the uniaxial anisotropy that enforces 180° domain walls. From the Lagrangian, it's easy to obtain

$$\boldsymbol{m} = \frac{S}{a} \boldsymbol{n} \times \dot{\boldsymbol{n}}. \tag{4}$$

By integrating out $\boldsymbol{m}$, the Lagrangian is reduced to

$$\mathcal{L} = \frac{\rho}{2}\dot{\boldsymbol{n}}^2 - s\boldsymbol{a}(\boldsymbol{n}) \cdot \dot{\boldsymbol{n}} - \mathcal{U}(\boldsymbol{n}), \tag{5}$$

where $\rho = \mathcal{S}^2/a$. Inserting the Lagrangian into Euler-Lagrange equation generates the dynamics of the staggered field $\boldsymbol{n}$

$$\rho \boldsymbol{n} \times \ddot{\boldsymbol{n}} + s\dot{\boldsymbol{n}} = \boldsymbol{n} \times \frac{\delta \mathcal{U}}{\delta \boldsymbol{n}}. \tag{6}$$

This equation is useful for producing domain wall dynamics and describing magnon excitation.

Structure of the Nèel domain wall

The static spin texture can be characterized by staggered field $\boldsymbol{n}$, which can be obtained by minimizing the energy $\mathcal{U}(\boldsymbol{n})$ with respect to $\boldsymbol{n}$. Parametrizing the staggered field by angle variables $\boldsymbol{n} = (\sin\theta\cos\phi, \sin\theta\sin\phi, \cos\theta)$, $\mathcal{U}(\boldsymbol{n})$ is rewritten as

$$\mathcal{U}(\boldsymbol{n}) \simeq \frac{A}{2}[(\boldsymbol{\nabla}\theta)^2 + \sin^2\theta(\boldsymbol{\nabla}\phi)^2] + \frac{K_e}{2}\cos^2\theta - \frac{K_{uni}}{2}\sin^2\theta\sin^2\phi. \tag{7}$$

Here, we drop the biaxial anisotropy based on the relevant energy scale hierarchy ($K_e \gg K_{uni} \gg K_c$), and choose the coordinate by aligning y-axis along the easy axis of uniaxial anisotropy $\boldsymbol{u}$. Upon minimizing the free energy with respect to θ and ϕ, the equation determining the magnetization profile reads

$$\begin{aligned} &-2A\theta'' + A\sin 2\theta(\phi')^2 - K_e \sin 2\theta - K_{uni}\sin 2\theta \sin^2\phi = 0, \\ &-2A\sin\theta(\sin\theta\phi'' + 2\cos\theta\theta'\phi') - K_{uni}\sin^2\theta\sin 2\phi = 0. \end{aligned} \tag{8}$$

Since $K_e \gg K_{uni}$, the spins in the ground state tend to lie in the easy plane, so we consider the solution with $\theta = \pi/2$, and ϕ satisfies

$$-2A\phi'' + \frac{1}{2}K_c \sin 4\phi - K_{uni}\sin 2\phi = 0. \tag{9}$$

To obtain an analytical solution, we neglect the the term proportional to K_c as $K_c \ll K_{uni}$, which leads to

$$\phi'' = -\frac{1}{\lambda^2}\sin\phi\cos\phi \tag{10}$$

where $\lambda = \sqrt{A/K_{uni}}$. The minus sign on the right hand side of the equation can be absorbed into variables by setting $\phi = \varphi - \pi/2$, which gives

$$\varphi'' = \sin\varphi\cos\varphi/\lambda^2. \tag{11}$$

By taking integral on both side, a useful relation is obtained $\varphi' = \sin\varphi/\lambda$. When a DW with positive topological charge is considered, this equation can be solved by

$$\cos\varphi = \tanh\frac{y-Y}{\lambda}, \qquad \sin\varphi = \mathrm{sech}\frac{y-Y}{\lambda}, \tag{12}$$

where Y is the position of the domain wall. This solution suggests $\varphi = 0$ or π for $y = \pm\infty$, or $\phi = \pm\pi/2$, namely a head-to-head or tail-to-tail 180° domain wall.

Spin waves and angular momentum transport

Reflectionless transmission of spin waves expected through static domain walls. Here, we show that a static domain wall should act only as a reflectionless Pöschl-Teller potential [7] for spin waves in a ferrimagnet, for wavelengths sufficiently short relative to the domain wall width.

Spin waves in a ferrimagnet can be described by small fluctuation of staggered field on top of the static spin texture. The fluctuation can be captured by variation of angle variables, i.e., $\theta \to \theta_0 + \delta\theta$, $\phi \to \phi_0 + \delta\phi$, where θ_0, ϕ_0 describe the domain wall profile discussed above. By substituting the angle variables containing small variation into Eq. (5), the spin wave Lagrangian is extracted as below

$$\mathcal{L}_{\mathrm{sw}} = \frac{\rho}{2}[(\partial_t\delta\theta)^2 + \sin^2\theta_0(\partial_t\delta\phi)^2] + s\delta\phi\partial_t\delta\theta - \frac{K_{uni}}{2}\delta\theta(\mathcal{D}+\kappa)\delta\theta - \frac{K_{uni}}{2}\delta\phi\mathcal{D}\delta\phi \tag{13}$$

where $\theta_0 = \pi/2$, $\mathcal{D} = -\lambda^2\partial_y^2 - 2\mathrm{sech}^2[(y-Y)/\lambda] + 1$ and $\kappa = K_e/K_{uni}$. The dynamic equations for fluctuations are

$$\begin{pmatrix} s\partial_t & -\rho\partial_t^2 - K_{uni}\mathcal{D} \\ -\rho\partial_t^2 - K_{uni}(\mathcal{D}+\kappa) & -s\partial_t \end{pmatrix}\begin{pmatrix}\delta\theta \\ \delta\phi\end{pmatrix} = 0. \tag{14}$$

Note that $\xi(q) = \frac{\tanh\tilde{y} - iq}{-iq-1}e^{iq\tilde{y}}$ is the eigenfunction of $\mathcal{D}$ with $\mathcal{D}\xi(q) = (1+q^2)\xi(q)$, where $\tilde{y} = (y-Y)/\lambda$ and $q = \lambda k$. Assuming $\delta\theta, \delta\phi \propto e^{i(ky-\omega t)}$, it is straightforward to gain

$$\omega_\pm^2 = \frac{1}{2\rho^2}[s^2 + K_{uni}\rho(2+2q^2+\kappa) \pm \sqrt{s^4 + 2\rho s^2 K_{uni}(2+2q^2+\kappa) + K_{uni}^2\kappa^2\rho^2}]. \tag{15}$$

When $\tilde{y} \gg 1$, $\xi(q) \sim e^{iky}$, the wavefunction approaches to the plane wave and frequency keeps unchanged. Here, these two branches of spin wave eigenstate are polarized to opposite direction along the staggered field $\boldsymbol{n}$. For a spin wave of a given branch, the polarization will flip direction after traveling through the domain wall, as shown in Supplementary Figure 12.

The discussion above is valid for magnons with wavelength $(2\pi/k)$ smaller than the domain wall width λ. For longer wavelengths, magnons can be significantly reflected by domain walls. In our system, given $A = 2.22\times10^{-12}$ J/m and $K_{uni} = 4\times10^3$ J/m^3, the domain wall width is $\lambda = \sqrt{A/K_{uni}} \simeq 24$ nm. So the critical momentum is given by

$$k_c = \frac{2\pi}{\lambda} \simeq 0.26\ \mathrm{nm}^{-1}, \tag{16}$$

and the reflectionless magnon picture is valid for $k > k_c$. The experiment is performed at room temperature, so the magnons with energy smaller than $k_B T_r$ with $T_r \simeq 300$ K are activated. To estimate the activated portion of magnon bands, we roughly neglect the difference between two bands by taking $s \to 0$ and $\kappa \to 0$ in Eq. (15) because the energy scale of band structure is ruled by exchange energy, so that $E(k) \sim \hbar\sqrt{(K_{uni} + Ak^2)/\rho}$. Here, $\rho = \mathcal{S}^2/a$ where $a = (2D/d^2)A$ and $\mathcal{S} \sim \hbar/d^3$ ($D = 3$ for three-dimensional system and $d \sim 1$ nm is the length of unit cell) [8]. Letting $E(k_m) \simeq k_B T_r$, we obtain an upper limit for k_m which defines the active region for magnon bands,

$$k_m \sim 0.8\ \mathrm{nm}^{-1}. \tag{17}$$

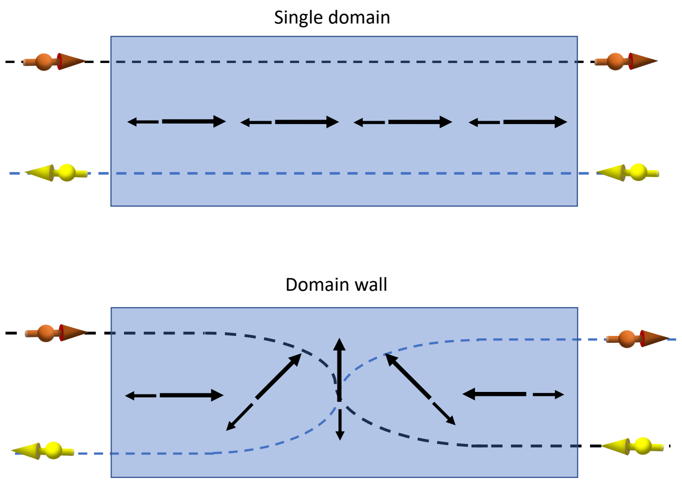

Supplementary Figure 12. In a single-domain ferrimagnet the two eigenmodes of magnons have opposite polarizations, and the polarizations stay unchanged over the sample. In the multi-domian case, the polarization of each eigenmode reverses adiabatically across a 180° domain wall.

Since the fraction of the reciprocal space with activated magnons for which $k > k_c$ is $(k_m/k_c)^3 \sim 30$, the reflectionless picture should be valid for most of the active magnons.

Spin wave coupling to domain wall dynamics. The calculation above neglects the dynamics of domain wall. However, if a magnon reverses its polarization direction upon passing through a domain wall, the change in angular momentum can be absorbed by the domain wall through a shift in its position. We speculate that even if a domain wall is on average pinned over long time scales, it may still be possible for the domain wall to absorb and then re-emit this angular momentum back into the magnon population rather than causing the change in angular momentum to be lost to the lattice. In principle, this dynamical shuttling of angular momentum from incident magnon to domain wall to a transmitted magnons could allow efficient transmission of angular momentum past a domain wall without reversal of the angular momentum.

[1] A. Vansteenkiste, J. Leliaert, M. Dvornik, M. Helsen, F. Garcia-Sanchez, and B. Van Waeyenberge, The design and verification of MuMax3, AIP advances **4**, 107133 (2014).
[2] S. C. P. van Kooten, *Ultrafast manipulation of magnetism in a Van der Waals ferromagnet*, Ph.D. thesis, Eindhoven University of Technology (2020).
[3] C. Kittel, Physical theory of ferromagnetic domains, Reviews of modern Physics **21**, 541 (1949).
[4] A. V. Azovtsev and N. A. Pertsev, Dynamical spin phenomena generated by longitudinal elastic waves traversing $CoFe_2O_4$ films and heterostructures, Phys. Rev. B **100**, 224405 (2019).
[5] S. Emori, D. Yi, S. Crossley, J. J. Wisser, P. P. Balakrishnan, B. Khodadadi, P. Shafer, C. Klewe, A. T. N'Diaye, B. T. Urwin, K. Mahalingam, B. M. Howe, H. Y. Hwang, E. Arenholz, and Y. Suzuki, Ultralow damping in nanometer-thick epitaxial spinel ferrite thin films, Nano Letters **18**, 4273 (2018).
[6] S.-H. Oh, S. K. Kim, J. Xiao, and K.-J. Lee, Bidirectional spin-wave-driven domain wall motion in ferrimagnets, Phys. Rev. B **100**, 174403 (2019).
[7] G. Pöschl and E. Teller, Bemerkungen zur quantenmechanik des anharmonischen oszillators, Zeitschrift für Physik **83**, 143 (1933).
[8] E. G. Tveten, T. Müller, J. Linder, and A. Brataas, Intrinsic magnetization of antiferromagnetic textures, Phys. Rev. B **93**, 104408 (2016).
